# CD138 and CD31 Double-Positive Cells Comprise the Functional Antibody-Secreting Plasma Cell Compartment in Primate Bone Marrow

**DOI:** 10.3389/fimmu.2016.00242

**Published:** 2016-06-27

**Authors:** Paola Martinez-Murillo, Lotta Pramanik, Christopher Sundling, Kjell Hultenby, Per Wretenberg, Mats Spångberg, Gunilla B. Karlsson Hedestam

**Affiliations:** ^1^Department of Microbiology, Tumor and Cell Biology, Karolinska Institutet, Stockholm, Sweden; ^2^Immunology Division, Garvan Institute of Medical Research, Darlinghurst, NSW, Australia; ^3^Division of Clinical Research Centre, Department of Laboratory Medicine, Karolinska Institutet, Huddinge, Sweden; ^4^Department of Orthopedic Surgery, Karolinska University Hospital, Stockholm, Sweden; ^5^Comparative Medicine, Astrid Fagraeus Laboratory, Karolinska Institutet, Stockholm, Sweden

**Keywords:** plasma cells, rhesus macaque, antibodies, elispot, bone marrow, cryopreservation

## Abstract

Plasma cells (PCs) are defined as terminally differentiated B cells that secrete large amounts of immunoglobulin (Ig). PCs that reside in the bone marrow (BM) are responsible for maintaining long-term antibody (Ab) responses after infection and vaccination, while PCs present in the blood are generally short-lived. In rhesus macaques, a species frequently used for the evaluation of human vaccines, B cells resemble those found in humans. However, a detailed characterization of BM-resident rhesus PC phenotype and function is lacking. Here, we examined Ig secretion of distinct rhesus CD138+ populations by B cell ELISpot analysis to couple phenotype with function. We demonstrate that the CD20low/−CD138+CD31+ BM population was highly enriched for antibody-secreting cells with IgG being the predominant isotype (60%), followed by IgA (33%) and IgM (7%). Transmission electron microscopy analysis confirmed PC enrichment in the CD20low/−CD138+CD31+ population with cells containing nuclei with “spokes of a wheel” chromatin structure and prominent rough endoplasmic reticulum. This panel also stained human BM PCs and allowed a clear distinction between BM PCs and short-lived peripheral PCs, providing an improved strategy to isolate PCs from rhesus BM for further analysis.

## Introduction

Durable immunity relies on constitutive antibody (Ab) production by terminally differentiated, long-lived bone marrow (BM)-resident plasma cells (PCs). These cells are products of the germinal center (GC) reaction where B cells proliferate upon recognition of cognate antigen in a T helper cell-dependent manner. Following somatic hypermutation (SHM) of Ab variable domains, selected B cells differentiate either into memory B cells or PCs prior to exiting the GC [reviewed in Ref. (1)]. Blimp-1, along with XBP-1 and IRF-4, is a key transcription factor for the transition to fully differentiated PCs (2).

In acute inflammatory situations, such as in response to infection or immunization, new peripheral PCs are generated. These cells are short-lived and migrate toward the inflammatory sites to secrete antigen-specific antibodies (3, 4). The BM is the main compartment where long-lived PCs persist in healthy individuals, and homing to this compartment is essential for long-term serological memory. This process is tightly regulated by the expression of adhesion molecules, chemokines and their receptors, where CXCL12 and its receptor, CXCR4, play major roles (5, 6). The BM provides a milieu that supports the persistence of long-lived PCs (7). Its cellular components consist mainly of CXCL12+VCAM+ stromal cells and hematopoietic cells (5, 8), including eosinophils and megakaryocytes, capable of secreting IL-6 and APRIL that regulate the frequency and survival of PCs *via* their receptors IL-6R and BCMA/TACI (9–11), respectively (9–12). BAFF and APRIL signaling upregulates the expression of the anti-apoptotic molecule Mcl-1, which is essential for the long-term survival of PCs (13). In addition, the B cell intrinsic program that is imprinted during the GC reaction by intrinsic factors is indispensable for determining the fate and maintaining durable Ab responses (12, 14).

In humans, BM-resident PCs are typically defined by the expression of CD38 and CD138 as well as heterogeneous CD19 expression (15), combined with their ability to secrete Ab or stain positively for cytoplasmic Ig. Recent studies reported that of these PCs, the CD19− cells display a more differentiated phenotype (16) that was associated with a longer life span (17) compared to CD19+ PCs. Less information is available about BM-resident PCs in non-human primates. Rhesus macaques are frequently used to model human immunology. With the publishing of the rhesus macaque genome (18) and the development of protocols to phenotype rhesus B cell populations and PCR amplify rhesus immunoglobulin (Ig) genes (19, 20), examination of B cell responses in this species is now more accessible.

Phenotypic characterization of PCs in rhesus macaques has primarily focused on the analysis of blood where plasmablasts are abundant 1 week following immunization (21, 22). Two recent reports describe phenotypic analysis of BM-resident PCs in macaques based on markers that work well for human BM PCs, including CD138, CD38, CD27, and/or CD19 (23, 24). However, as these markers are also expressed on other cell types and their pattern of expression may be different in macaques, efforts to characterize additional markers that distinguish rhesus macaque BM PCs are needed. Here, we describe a comprehensive phenotypic and functional characterization of rhesus macaque BM PCs. Specifically; we show that antibody-secreting cells (ASC) are contained within the CD20low/−CD138+CD31+ population. This staining panel discriminated BM PCs from peripheral plasmablasts and was also suitable for staining human BM PCs. We show further that the CD19 and CD38 markers, often used to define human BM PCs, are suboptimal for defining rhesus macaque BM PCs due to reduced cross-reactivity and/or different expression patterns. Finally, we show that cryopreservation of rhesus BM cells led to a selective loss of the CD20low/−CD138+CD31+ population, which has practical implications for studies of BM PCs.

## Materials and Methods

### Ethics Statement

The animal work was conducted with the approval of the regional Ethical Committee on Animal Experiments (Stockholms Norra Djurförsöksetiska Nämnd), while the human work was conducted with the approval of the regional ethical vetting board in Stockholm with the registration number 2015/305-31/1. All methods were carried out in accordance with the approved guidelines.

### Animals

Rhesus macaques (*Macaca mulatta*) of Chinese origin, ~5–6 years old, were housed at the Astrid Fagraeus Laboratory facility at Karolinska Institutet, as previously described (21, 25). Housing and care procedures were in compliance with the provisions and general guidelines of the Swedish Board of Agriculture, and the facility has been assigned an Animal Welfare Assurance number by the Office of Laboratory Animal Welfare (OLAW) at the National Institutes of Health (NIH). The macaques were housed in pairs in 4 m^3^ cages, enriched to give them the possibility to express their physiological and behavioral needs. They were habituated to the housing conditions for more than 6 weeks before the start of the experiment and subjected to positive reinforcement training in order to reduce the stress associated with experimental procedures. All immunizations and blood samplings were performed under sedation with ketamine 10–15 mg/kg intramuscularly (i.m.) (Ketaminol 100 mg/ml, Intervet, Sweden). BM aspirates were collected as described previously (26). The macaques were weighed at each sampling. All animals were confirmed negative for simian immunodeficiency virus (SIV), simian T-cell lymphotropic virus, simian retrovirus type D, and simian Herpes B virus.

### Human Subjects

Bone marrow cells from three adult human subjects were acquired in conjunction with hip-replacement surgery at the Orthopedics Clinic, Karolinska University Hospital, in Stockholm. Oral and written consent was obtained.

### Isolation and Cryopreservation of Mononuclear Cells from Blood and Bone Marrow

Mononuclear cells from peripheral blood and BM were isolated within 2 h of sample collection using BD Vacutainer^®^ K2 EDTA tubes. Tubes were immediately inverted 8–10 times, and blood tubes were centrifuged at 15 min at 2400 rpm at 25°C to separate the plasma. Blood or BM cells were diluted in sterile PBS and layered on top of Ficoll-Paque™ (GE Healthcare Life Sciences) at a 2:1 ratio, and the tubes were centrifuged at 30 min at 2200 rpm at 25°C without break. A 5 ml sterile serological pipette was used to isolate the buffy coat layer in a 50 ml conical tube. The cells were washed twice with PBS (1750 rpm, 10 min, 25°C), 3 ml of lysis buffer was added for 5 min, and cells were washed twice with PBS (1500 rpm, 10 min, 25°C) and resuspended in 5 ml of PBS with 5% FBS (Sigma) per tube of blood collected. The cells were counted using 0.4% trypan blue exclusion countess (Life Technologies). An aliquot of the cells was used fresh in ELISpot assays, and the remaining cells were frozen at 10–15 × 10^6^/ml/cryovial in Fetal Bovine Serum (FBS) with 10% DMSO chilled at 4°C. Cryovials containing cells were placed in an ice bath for 15 min prior to placing in a “Mr. Frosty” (Nalgene) cryocontainer before transfer, first to −80°C for 24 h and then, to liquid nitrogen tanks. To thaw the cells, the cryovials were removed from the liquid nitrogen and brought on dry ice to the lab. Vials were thawed immediately while shaking in a 37°C water bath until a few ice crystals remained. No more than one cryovial was thawed at the same time. The cell suspension was transferred into a 10 ml of pre-warmed RPMI 1640 media (Sigma), supplemented 10% FCS (Sigma), 2 mM l-glutamine, 100 U/ml penicillin, 100 mM streptomycin, and 2% HEPES (supplemented media), all from Life Technologies. The tubes were centrifuged at 1500 rpm for 5 min with the brake set at low speed. After one additional wash in supplemented media, the cells were resuspended in 5 ml of PBS. Cells were counted and viability assessed using 0.4% trypan blue exclusion. The viability typically ranged from 85 to 95%.

### Flow Cytometry of BM PCs

Frozen BM cells were thawed at 37°C and washed twice in pre-warmed supplemented media. Cells were washed twice with PBS and counted using 0.4% trypan blue exclusion countess (Life Technologies), and the cell viability was consistently >90%. Cells were resuspended in 100 μl of PBS in a 96-well plate (1–4 × 10^6^ cells/well) and incubated for 10 min at 4°C with FcR blocking (eBioscience or BD) for rhesus and human cells respectively, and Aqua Dead Cell Stain Kit (Molecular Probes). Cells were washed with FACS buffer (PBS + 2% FBS). Rhesus cells were surface-stained with antibodies against CD3 clone SP34-2 BV786 (BD), CD20 clone 2H7 BV421 (BD), CD138 clone DL101 PE (eBioscience), CD49d clone 9F10 PerCp-Cy5.5 (eBioscience), CD31 clone WM59 Alexa 647 (BD), CD98 clone 5E5 FITC (eBioscience), CD19 clone J3-119 PE-Cy7 (Beckman Coulter), and CD38 clone OKT10 APC (NIH Non-human primate reagent resource). Human cells were surface-stained using the same Ab panel as used for macaques with the exception of CD38 [clone HIT2 APC (BD)]. All Abs were previously titrated for optimal staining of rhesus macaque and human peripheral blood mononuclear cells (PBMCs). Samples were collected on a FACS LSRII or sorted using a FACS ARIA FUSION (BD Immunocytometry Systems) and analyzed using FlowJo software.

### Flow Cytometry of Circulating Plasmablasts in Rhesus Macaque Blood

Frozen PBMCs were thawed, and cell viability was consistently >90%. After the incubation with the FcR blocking (BD) and Aqua Dead Cell Stain (Molecular Probes), as described in Section “Flow Cytometry of BM PCs,” the cells were washed with FACS buffer and surface-stained for 15 min at 4°C with antibodies against CD3 BV786 (BD), CD20 BV421 (BD), CD138 PE (eBioscience), CD80 clone L307.4 APC-H7 (BD), HLA-DR clone L243 PerCP-Cy5.5 (BD), CD14 clone M5E2 BV786 (BD), CD16 clone 3G8 BV786 (BD), CD98 FITC (eBioscience), and CD49d APC (BD) or CD31 Alexa 647 (BD). Cells were washed twice with FACS buffer and incubated in 100 μl of Cytofix/Cytoperm (BD) for 20 min at 4°C. Cells were then washed twice in Perm/Wash buffer (BD) and intracellularly stained in 100 μl of Perm/Wash buffer for 15 min at 4°C with IgG clone G18-145 PE-Cy7 (BD), followed by two washes in Perm/Wash buffer and two in FACS buffer. All Abs were previously titrated for optimal staining of rhesus macaque PBMCs. Samples were collected on a FACS LSRII (BD Immunocytometry Systems) and analyzed using FlowJo software.

### B Cell ELISpot Assay

Plasma cells were enumerated as previously described (21). In brief, to evaluate the presence of BM PCs, defined as spontaneous ASC, different gate populations were sorted into supplemented media and plated directly into ELISpot plates. To detect ASC, MAIPSWU10 96-well plates (Millipore) were coated with 10 μg/ml antihuman IgG, IgA, and IgM (Fcγ, Fcα, and Fcμ chain) (Jackson ImmunoResearch), and cells were transferred to the plates in dilution series and incubated for 18 h at 37°C, 5% CO_2_. The plates were then washed with PBS containing 0.05% Tween and incubated with biotinylated antihuman IgG, IgA, or IgM (0.25 μg/ml) for 1.5 h at 37°C followed by washing and incubation with streptavidin-AP (1:1000, Mabtech). The reactions were developed using BCIP/NBT substrate (Sigma) and stopped by washing in distilled water. Spots corresponding to ASC were counted using an ImmunoSpot^®^ analyzer (Cellular Technology Ltd.). The results were converted to show percentage of ASC of plated events.

### Transmission Electron Microscopy

CD138+CD31+ cells were sorted from a frozen sample, and 34,000 events were acquired into supplemented media. The cells were washed twice with PBS before fixing in 2.5% glutaraldehyde in 0.1M phosphate buffer, pH 7.4 at +4°C overnight, and centrifuged to a pellet. The pellet was rinsed in 0.1M phosphate buffer, pH 7.4. A small amount of 10% gelatine was added on top of the small pellet and left to solidify. The pellet was then fixed in 2% osmium tetroxide in 0.1M phosphate buffer, pH 7.4 at +4°C for 2 h, dehydrated in ethanol followed by acetone and embedded in LX-112 (Ladd, Burlington, VT, USA). Ultrathin sections (~50–60 nm) were cut using a Leica EM UC 6 (Leica, Wien, Austria). Sections were contrasted with uranyl acetate followed by lead citrate and visualized in a Hitachi HT 7700 (Tokyo, Japan) at 80 kV. Digital images were taken by using a Veleta camera (Olympus Soft Imaging Solutions, GmbH, Münster, Germany).

### Statistical Analysis

All statistical analysis was done with GraphPad Prism software version 6 and considered significant at *p ≤ 0.05, **p ≤ 0.01, ***p ≤ 0.001, and ****p ≤ 0.0001. Comparisons of ≥3 groups were analyzed by the ANOVA non-parametric Kruskal–Wallis test followed by Dunn’s post test for individual comparisons. For repetitive measures, ANOVA was used. When comparing two groups, *t*-test or Mann–Whitney test were used. To analyze groups with multiple factors, two-way ANOVA was used followed by Bonferroni’s post test for multiple comparisons. The relationship between variables was established with the non-parametric Spearman’s correlation analysis.

## Results

### CD3−CD20low/−CD138+ Bone Marrow Cells in Rhesus Macaques Are Enriched for IgG-Secreting Cells

Bone marrow-resident PCs in humans are phenotypically defined as CD3 negative cells that have downregulated their surface expression of CD20 and express high levels of CD38 and CD138. Functionally, the hallmark of PCs is their capacity to secrete Ab constitutively both *in vivo* and when cultured *in vitro*. In rhesus macaques, which closely mimic humans both genetically and in regards to immune cell phenotypes, BM PCs were defined as CD20−CD19+CD38+CD138+ cells (23, 24). While this provides a useful starting point, it was shown that the CD19+CD20− population in cynomolgus macaques contained rhesus B-1-like B cells (27), and in humans, both CD19+ and CD19− populations harbor BM PCs (16, 17). Furthermore, currently available CD19 antibodies stain rhesus CD19 suboptimally (Figure S1 in Supplementary Material); thus, the definition of additional markers of rhesus BM PCs coupled with functional analysis is needed for improved definition of these cells.

**Figure 1:**
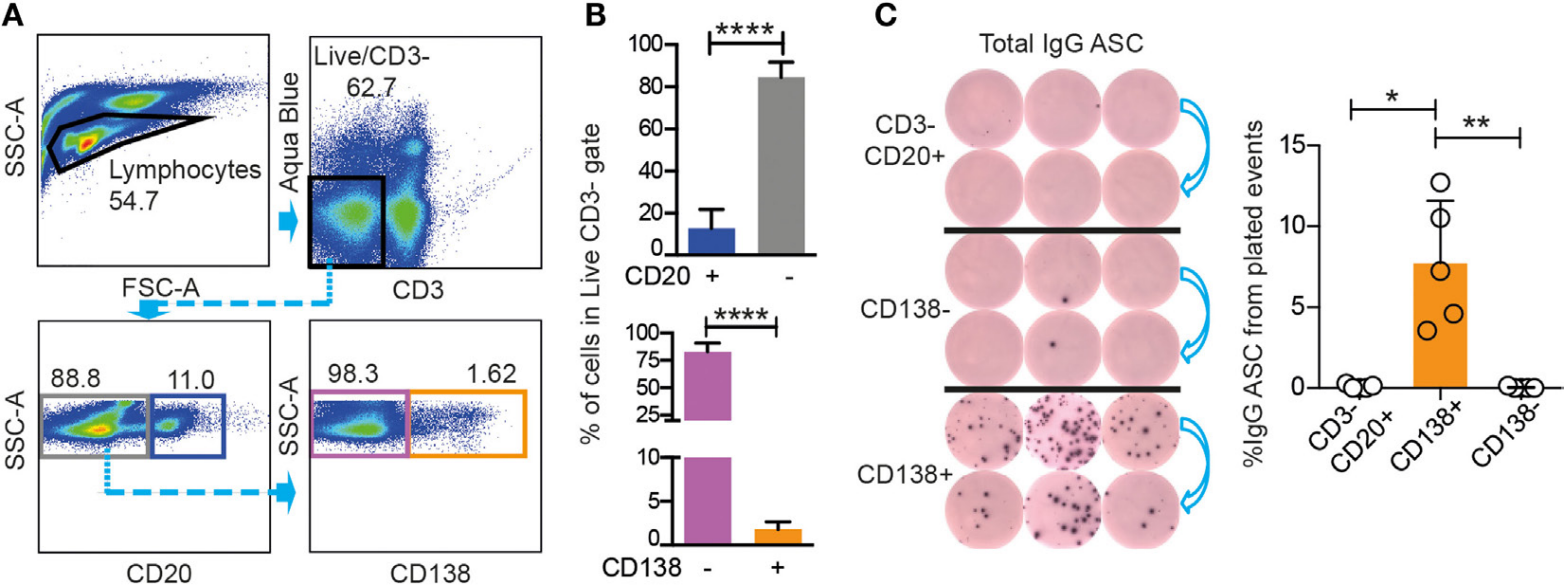
**Rhesus BM IgG ASC are found in the CD20low/−CD3−CD138+ population**. **(A)** FACS gating strategies for CD20+ (blue), CD20− (gray) populations gated from live, CD3− cells, and for CD138+ (orange) and CD138− (purple) populations gated from live, CD3−CD20low/− rhesus cells. **(B)** Frequency (mean and SD) of rhesus CD20+, CD20−, and CD138+, CD138− cells from the live, CD3−. Mann–Whitney test was done ****p < 0.0001 (*n* = 9). **(C)** ELISpot for total IgG ASC from the sorted populations shown in **(A)** are CD138+ (orange sort gate), CD138− (purple sort gate), and CD3−CD20+ (gray sort gate). Data from three different animals are shown, and the sorted cells were plated in two different dilutions (left panel). Frequency (mean and SD) of total IgG ASC in the three sorted populations are labeled as: CD138+, CD138−, and CD3−CD20+. Kruskal–Wallis test followed by Dunn’s multiple comparison were performed *p < 0.05 and **p < 0.01 (*n* = 5). All data were obtained from frozen samples.

To meet this objective, we first examined if CD3−CD20low/−CD138+ BM cells could be functionally defined as Ab-secreting PCs (23, 24). We stained Ficoll-separated BM cells from nine rhesus macaques for CD3, CD20, and CD138. The analysis of one representative animal is shown as dot plots (Figure 1A), and data from all nine macaques are shown as mean values (Figure 1B). The mean frequency of CD3−CD20+ cells of live cells was 15 ± 7%, while the mean frequency of CD3−CD20low/− cells in the live gate was 85 ± 7%. To stain for CD138, we used the DL101 Ab clone, which previously was demonstrated to be cross-reactive with rhesus macaque CD138 (22–24). Staining of the LiveCD3−CD20low/− population resulted in distinct CD138− and CD138+ populations with 1.62% of the LiveCD3−CD20low/− cells being CD138+ in the representative animal (Figure 1A), and the mean frequency of CD138+ cells of the LiveCD3− cells for the nine macaques was 1.8 ± 0.8% (Figure 1B). We next wished to couple phenotype to function and sorted three populations of cells, LiveCD3−CD20+ cells, LiveCD3−CD20low/−CD138− cells, and LiveCD3−CD20low/−CD138+ cells, from three macaques. The cells were sorted directly into ELISpot plates coated with anti-IgG Ab and incubated for 18 h without additional stimulation to assess their capacity to constitutively produce IgG. IgG-secreting PCs were observed in the CD3−CD20low/−CD138+ population (8 ± 4%), while virtually no IgG ASC were observed in the other two populations CD3−CD20+ (0.08 ± 0.1%) and CD3−CD20low/−CD138− (0.03 ± 0.06%), respectively (Figure 1C). Considering that only 8% of CD3−CD20low/−CD138+ BM cells produced IgG, it appeared likely that a large proportion of the CD138+ cells were not functional PCs. Therefore, we examined the CD138+ cells for additional markers that would allow for an improved enrichment of Ab-secreting BM cells.

### IgG-, IgA-, and IgM-Secreting BM PCs Are Contained Within the CD138+CD31+ Population

**Figure 2:**
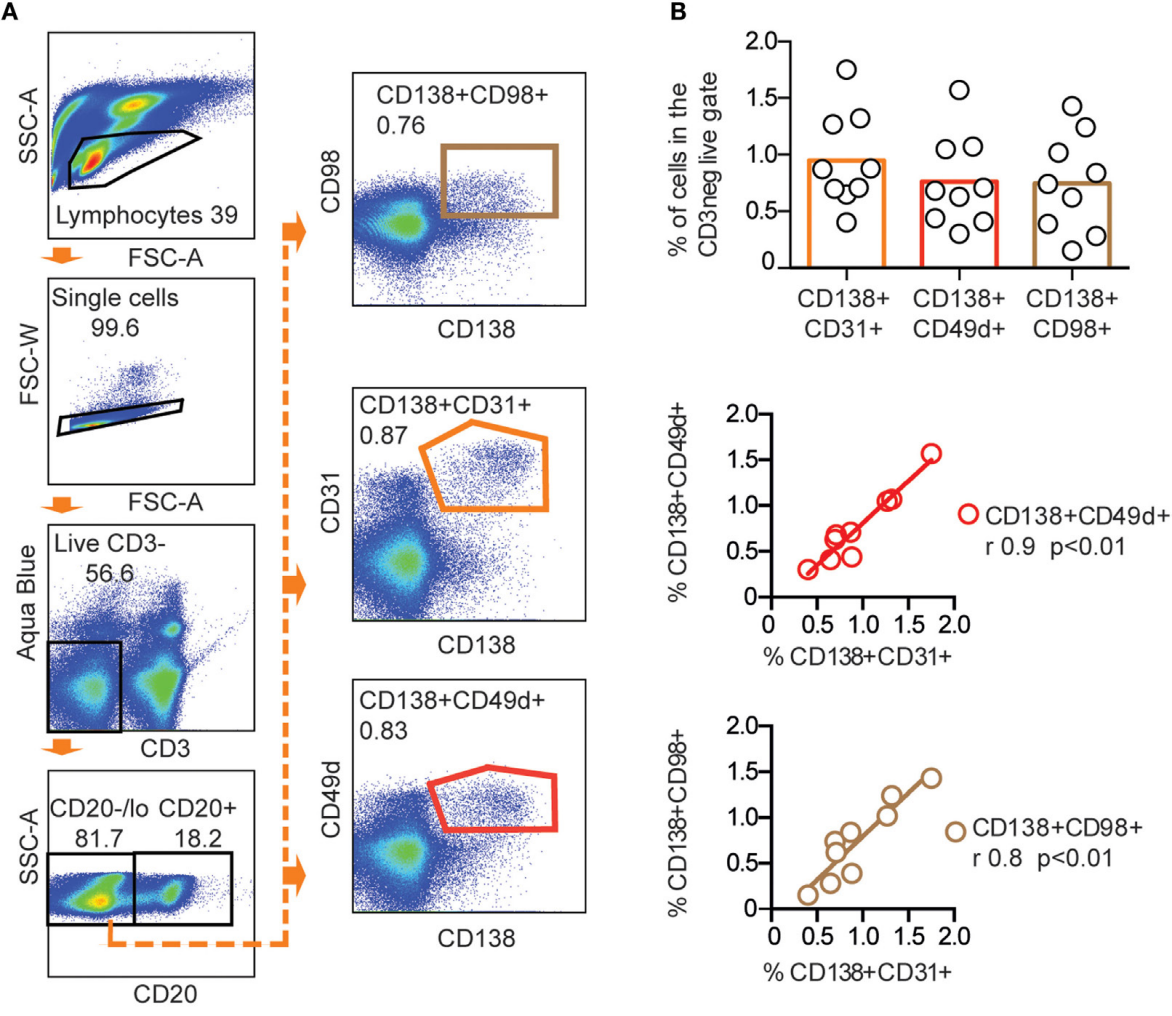
**CD31, CD49d, and CD98 expression of CD138+ rhesus bone marrow cells**. **(A)** Representative FACS plots showing CD138+CD31+, CD138+CD49d+, and CD138+CD98+ populations gated from live, CD3−CD20low/− cells in the same stain of rhesus bone marrow. **(B)** Frequency (mean) of CD138+CD31+, CD138+CD49d+, and CD138+CD98+ populations (upper panel). Correlations between CD138+CD31+, CD138+CD49d+, and CD138+CD98+ populations were analyzed using non-parametric Spearman (*r*) (**p < 0.01) and linear regression (middle and lower panels) *n* = 9. All data were obtained from frozen samples.

To identify additional markers that could enrich rhesus macaque BM PCs, we selected three molecules that were previously reported to be expressed by human or mouse PCs: CD49d, shown to be highly expressed on human BM PCs (15, 28), CD31, reported to be expressed on PCs in human spleen and BM (29), and CD98, which was recently suggested to be a marker of mouse PCs (30). We stained rhesus macaque CD138+ BM cells for CD31, CD49d, and CD98 and found that all three markers co-stained a subset of the CD138+ cells (Figure 2A). There was no statistically significant difference between the CD138+CD31+ (0.95 ± 0.4%), CD138+CD49d+ (0.8 ± 0.4%), and CD138+CD98+ (0.7 ± 0.4%) populations. We observed a high correlation in the frequencies between the CD138+CD31+ population, the CD138+CD49d+ (*r* = 0.9, **p < 0.01), and the CD138+CD98+ (*r* = 0.8, **p < 0.01) population (Figure 2B), which suggests that CD31, CD49d, and CD98 stained the same population. Therefore, we focused on one of the markers, CD31, for the subsequent analysis.

**Figure 3:**
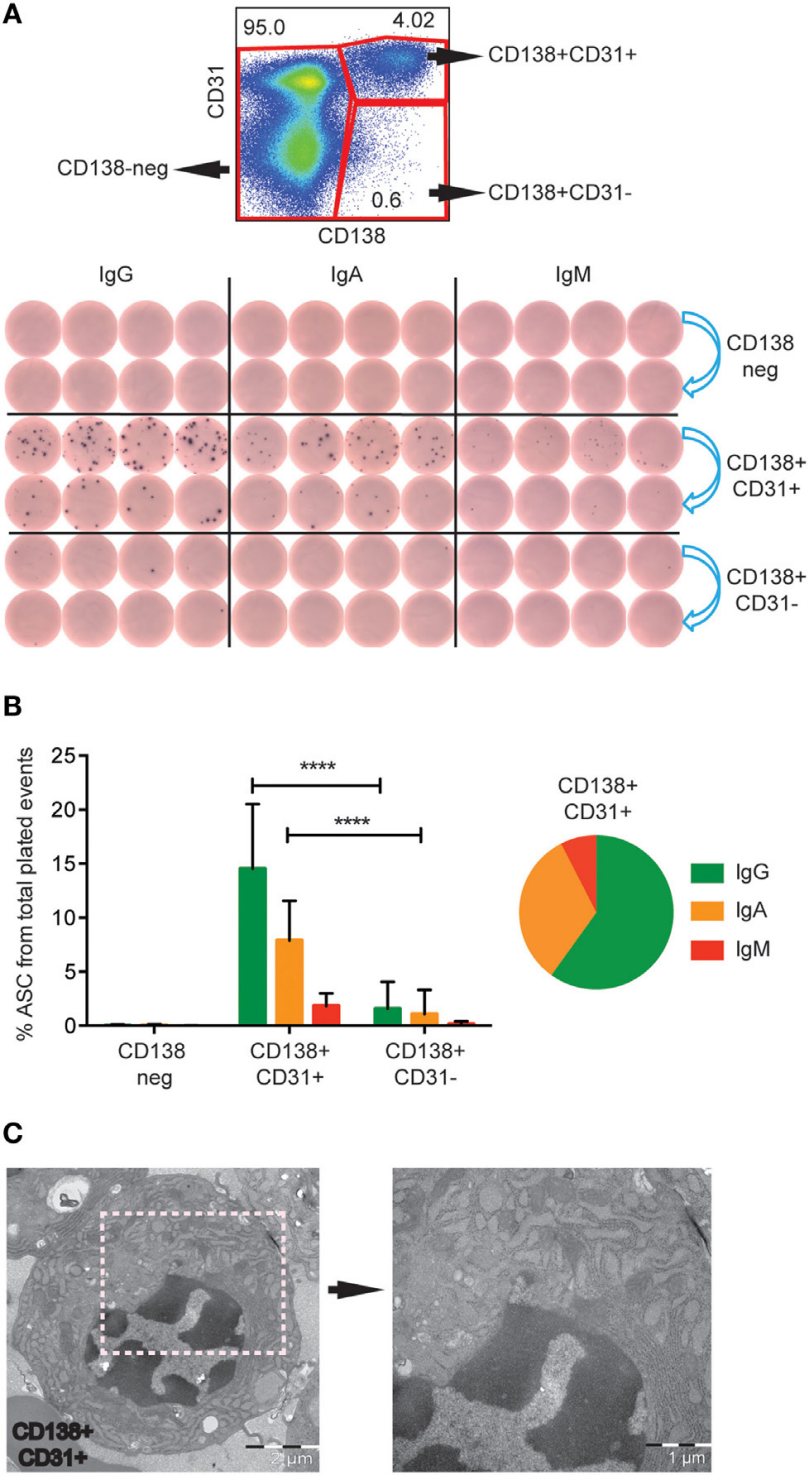
**IgG-, IgA- and IgM-secreting PCs are found in the CD138+CD31+ population**. **(A)** Representative FACS plot showing the gates of the three sorted populations (red gates), CD138+CD31+, CD138+CD31−, and CD138− (upper panel). Representative B cell ELISpot data of three experiments showing the sorted populations tested for IgG, IgA, and IgM secretion. 2000 events were sorted from each population, 2 dilutions of cells were plated at fourfold dilutions (blue arrows) (lower panel). **(B)** Frequencies (mean and SD) of IgG (green), IgA (orange), and IgM (red) ASC of total plated events from the three sorted populations. Pie chart of the CD138+CD31+ population, 60% of the cells secreted IgG (green), 32.5% IgA (orange), and 7.5% IgM (red). Data were obtained from fresh rhesus BM (*n* = 12). Two-way ANOVA followed by Tukey’s multiple comparison test was performed, ****p < 0.0001. **(C)** TEM analysis of cells sorted from the CD138+CD31+ population, exhibiting characteristic plasma cell morphology with typical “spokes of a wheel” chromatin structures, and prominent and dilated RER. Two different magnifications are shown with scale bars of 2 μm (left panel) and 1 μm (right panel). **(A,B)** data were obtained from fresh samples. **(C)** data were obtained from frozen samples.

To determine which population harbored Ab-secreting PCs, we next sorted LiveCD3−CD20low/−CD138+CD31+ and LiveCD3−CD20low/−CD138+CD31− cells from fresh rhesus BM into anti-IgG-, anti-IgA-, or anti-IgM-coated ELISpot plates. LiveCD3−CD20low/−CD138− cells were included as a negative control (Figure 3A, upper panel). Only the double-positive CD138+CD31+ population was significantly enriched for ASC (Figure 3A, lower panel). Combining the results obtained from 12 animals, we found that 24% of the plated CD138+CD31+ cells produced antibodies with the majority being of IgG isotype (14.5 ± 6.0%), followed by IgA (7.9 ± 3.7%) and IgM (1.8 ± 1.2%) (Figure 3B). These findings are consistent with studies of human BM PCs (16). When the plated CD138+CD31− cells were similarly analyzed, we found that 3% of the plated cells secreted antibodies with no significant difference between the IgG- (1.6 ± 2.5%), IgA- (1.1 ± 2.2%), and IgM-secreting (0.2 ± 0.2%) cells (Figure 3B). To assess PC enrichment in the sorted CD138+CD31+ population by morphological analysis, we performed Transmission Electron Microscopy (TEM). We found that PCs were the predominant cell type in these specimens, as distinguished by characteristic PC morphology, including nuclei with “spokes of a wheel” chromatin structure and prominent rough ER in the majority of the cells (Figure 3C). Altogether, these results illustrate that the CD138+CD31+ rhesus BM population comprises the functional IgG-, IgA-, and IgM-secreting PC compartment.

### Co-expression of CD138, CD31, CD49d, and CD98 Distinguishes BM Plasma Cells from Peripheral Plasma Cells

**Figure 4:**
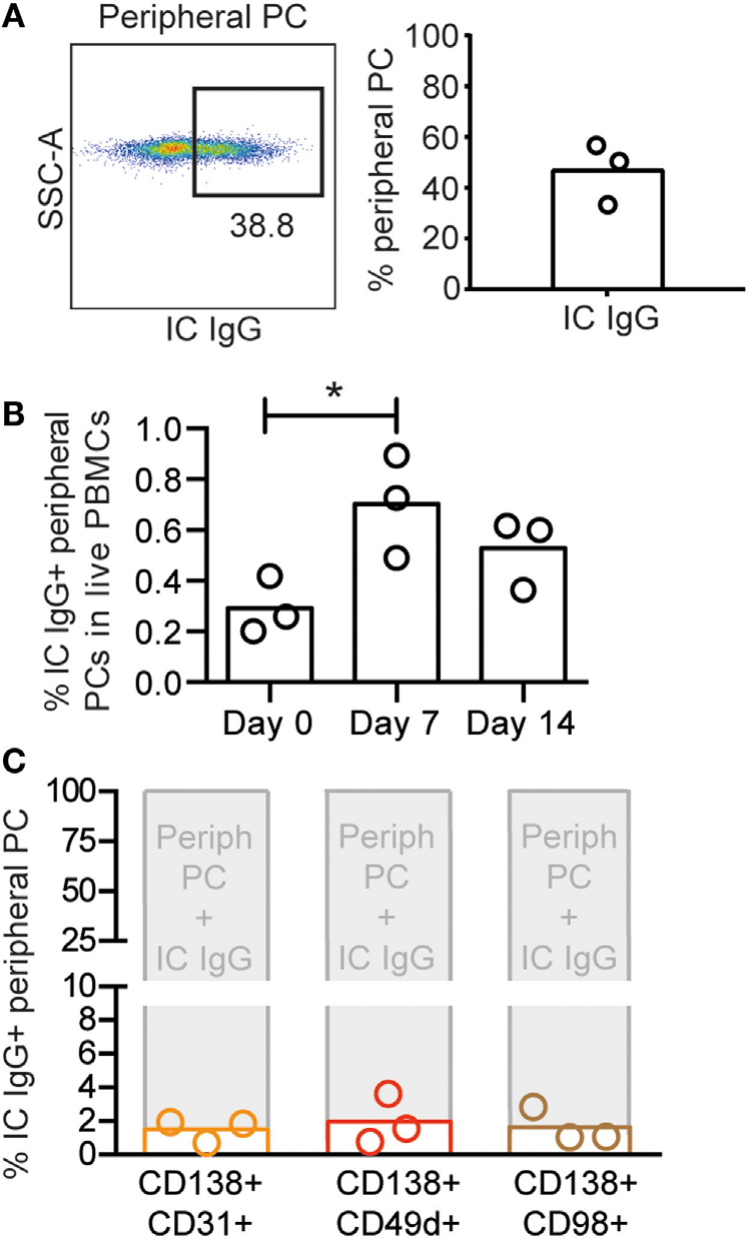
**Circulating plasmablasts in rhesus macaques are phenotypically distinct from BM PCs**. **(A)** Representative intracellular (IC) IgG plot (left panel) of rhesus peripheral PCs and percentages IC IgG+ cells of CD14−CD16−CD20low/−HLA-DR+CD80+ cells from day 7 postimmunization (*n* = 3) (right panel). **(B)** Frequency of IC IgG+ peripheral PCs in rhesus PBMCs collected on days 0, 7, and 14 postimmunization. To determine statistical significance, *p < 0.05, ANOVA was used for repetitive measures followed by Holm–Sidak’s multiple comparison **(C)** Percentages of CD138+CD31+ (orange), CD138+CD49d+ (red), and CD138+CD98+ (brown) populations (defined in Figure S2B in Supplementary Material) in IC IgG+ peripheral PCs. Each bar represents the mean, and each circle represents one animal (*n* = 3). All data were obtained from frozen samples.

We next sought to examine if the expression pattern of CD138, CD31, CD49d, and CD98 on blood PCs was similar to the one found in BM PCs. Circulating rhesus plasmablasts peaking 1 week after immunization were recently defined as CD3−CD16−CD20−/intHLA-DR+CD14−CD11c−CD123−CD80+ cells (22). We used the same staining panel with the exception of CD11c and CD123 (Figure S2A in Supplementary Material). To determine the percentage of these cells that co-stained with IC IgG, we examined samples collected 7 days after vaccination from a previous study (21). We found that 47 ± 14% of the LiveCD3−CD16−CD14−CD20−/intHLA-DR+CD80+ were positive for IC IgG (Figure 4A). Thus, plasmablasts were defined as LiveCD3−CD16−CD14−CD20−/intHLA-DR+CD80+IC IgG+ cells. Analysis of PBMCs collected 0, 7, or 14 days after vaccine inoculation (21) corroborated the occurrence of the peak peripheral PC response 7 days after immunization (*p < 0.05, ANOVA for repetitive measures) (Figure 4B). We next examined if CD14−CD16−CD20low/−HLA-DR+CD80 + IC IgG+ cells from the 7-day time point were CD138+CD31+, CD138+CD49d+ or CD138+CD98 and found negligible co-expression (Figure 4C) but rather single expression of CD31, CD49d, and CD98 due to the absence of CD138 expression on peripheral PCs (Figure S2B in Supplementary Material), in stark contrast to the phenotype of BM-resident PCs that display high expression of both CD138 and CD31.

### Human and Macaque BM PCs Similarly Co-express CD138 and CD31

**Figure 5:**
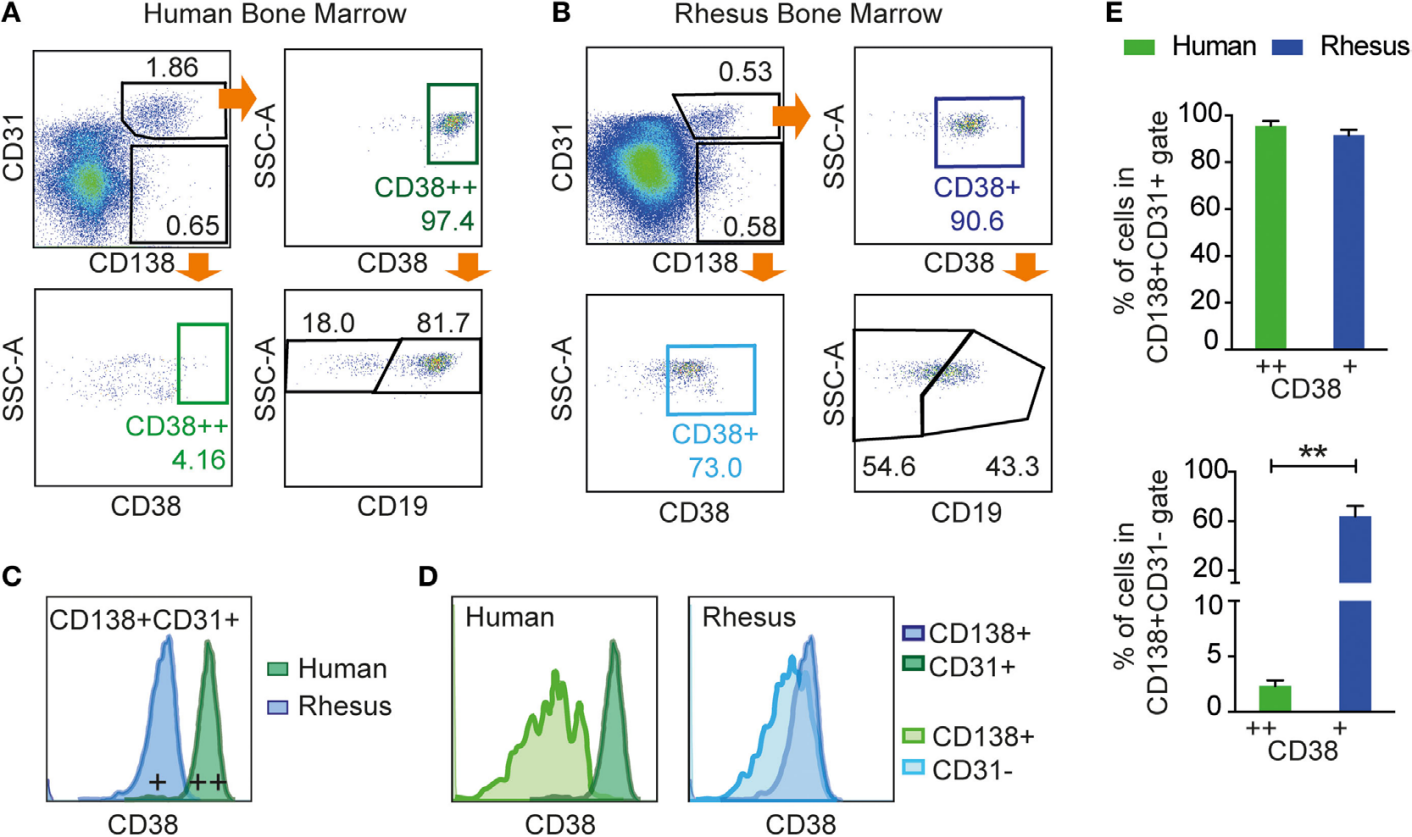
**Examination of CD19 and CD38 expression on human and rhesus CD3−CD20low/−CD138+CD31+ BM cells**. Flow cytometric analysis of **(A)** human BM and **(B)** rhesus BM using the CD138+CD31+ gating strategy with the addition of CD38 and CD19. CD138+CD31+ cells were gated from live CD3−CD20low/−. **(C)** Representative histogram of CD38 expression showing that CD138+CD31+ cells co-expressed CD38 in both human and rhesus BM, but with lower staining intensity in rhesus BM. **(D)** Representative histograms of the CD38 marker showing the separation of CD138+CD31+ from the CD138+CD31− population in human cells (dark and light green, respectively) and in rhesus cells (dark and light blue, respectively). **(E)** Percentages of CD38+ cells within the CD138+CD31+ population (top panel) and CD138+CD31− population (bottom panel) for human cells (green) and rhesus cells (blue). Unpaired *t*-tests were done using Welch’s correction to determine statistical significance, **p < 0.01. Bars and error bars represent mean and SD, respectively (*n* = 3). All data were obtained from frozen samples.

Having established that rhesus BM PCs are contained within the CD138+CD31+ population, we performed a direct comparison between macaque and human BM to determine if the same staining panel was suitable for identification of human BM PCs. The purpose of this was to perform a qualitative rather than quantitative comparison of PC populations in these hosts as the number of available human subjects was low. As mentioned earlier, CD38 and CD19 are used to define different subsets of human BM PCs. Hence, antibodies against these markers were added to the rhesus PC-staining panel, and BM samples from three human donors and three rhesus macaques were examined (Figures 5A,B). We clearly distinguished a CD138+CD31+ human BM PC population that also expressed high levels of CD38, while the same staining applied to rhesus macaque BM PCs gave only a dim CD38 signal (Figure 5C). Furthermore, CD38 expression robustly separated the human CD138+CD31+ and CD138+CD31− populations, while this was not the case for the corresponding rhesus BM cells (Figure 5D).

The distribution of CD138+CD31+ and CD138+CD31− populations was similar in rhesus and human BM samples. However, while CD138+CD31+ cells were positive for CD38 in both humans (95.6 ± 2.3% of cells defined as CD38++) and macaques (91.5 ± 2.8% of cells defined as CD38+) (Figure 5E, left), the CD138+CD31− population (Figure 5E, right) was negative for CD38 in humans (2.3 ± 0.5%), while a significant proportion of the rhesus macaque CD138+CD31− cells (63.9 ± 8.9%, **p < 0.01) expressed CD38 to a similar intensity as the CD138+CD31+ population. Additionally, we evaluated CD19 expression in human CD138+CD31+CD38++ population and in rhesus CD138+CD31+CD38+ population, and we found that both CD19+ and CD19− populations were clearly distinguishable on human cells but less so on rhesus cells (Figures 5A,B). Thus, the CD3−CD20low/−CD138+CD31+ staining panel identifies PCs in both human and rhesus BM, while markers frequently used to define human PCs, CD38 and CD19, are suboptimal for staining rhesus macaque BM PCs.

### Cryopreservation of Macaque Bone Marrow Cells Results in a Selective Loss of CD138+CD31+ Cells

**Figure 6:**
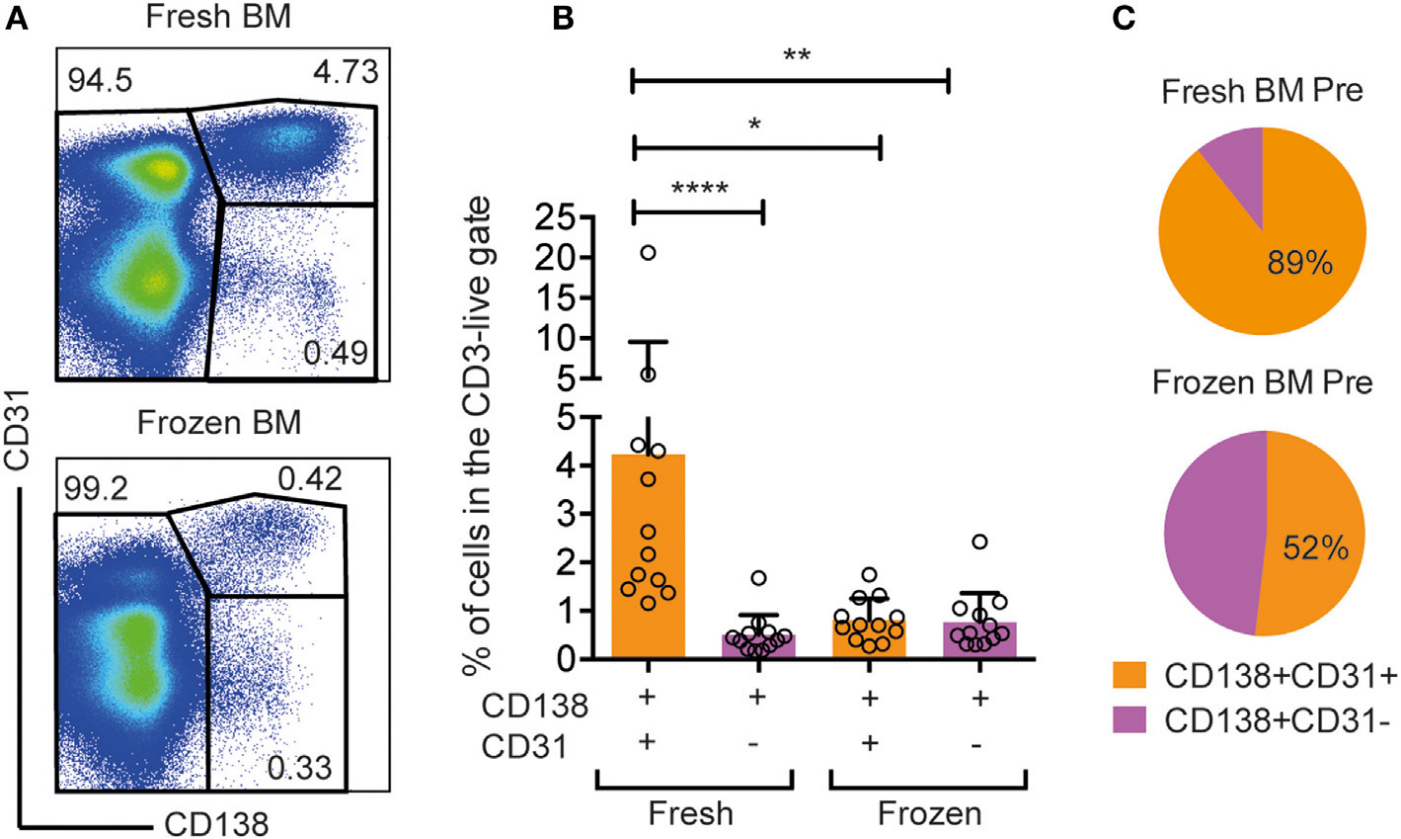
**Selective loss of CD138+CD31+ cells in frozen rhesus bone marrow samples**. **(A)** Representative flow cytometry dot plots of CD138+CD31+, CD138+CD31−, and CD138− populations gated from live, CD3−CD20low/− cells isolated from fresh and frozen bone marrow. **(B)** Frequencies of CD138+CD31+ (orange) and CD138+CD31− (purple) cells gated from live, single CD3−CD20low/−cells. Kruskal–Wallis test followed by Dunn’s multiple comparison test for non-parametric data was performed (*n* = 12). Significant differences were determined as *p < 0.05, **p < 0.01, and ****p < 0.0001. **(C)** Pie charts showing percentages of CD138+CD31+ (orange) and CD138+CD31− (purple) populations in frozen and fresh samples.

Rhesus macaques are widely used to evaluate immune responses induced by vaccine candidates under development for human clinical use. In most of these immunization studies, samples are collected and immediately frozen for subsequent analysis. We observed a striking difference in the frequency of CD138+CD31+ cells between fresh and frozen rhesus macaque BM cells (Figure 6). When staining fresh and frozen BM from a representative animal using the same gating, the frequency of CD138+CD31+ population in the LiveCD3−CD20low/− gate was 10 times higher in the fresh sample (4.73%) than in the frozen sample (0.42%) (Figure 6A). When extending our analysis to BM samples from 12 rhesus macaques, we observed a significantly higher frequency of CD138+CD31+ cells in fresh samples (4.2 ± 5.3%) than in the frozen samples (0.8 ± 0.4%) (Figures 6B,C) (*p < 0.05, Dunn’s multiple comparison test). The frequency of CD138+CD31− cells was similar in fresh and frozen samples indicating a selective loss of CD138+CD31+ PCs following freezing (Figures 6B,C). The selective loss of CD138+CD31+ after the cryopreservation highlights the advantage of working with fresh BM samples, which is relevant for studies that aim to analyze Ab-secreting PCs in this compartment.

## Discussion

The induction of persistent Ab responses is the goal of most prophylactic vaccines. Although rhesus macaques are frequently used for preclinical evaluation of human vaccine candidates, a comprehensive analysis of the phenotypic and functional properties of rhesus macaque BM PCs is lacking. Here, we demonstrate that IgG-, IgA-, and IgM-secreting rhesus BM PCs were contained within the CD31+ population of the CD20low/−CD138+ cells.

CD31 stains both BM PCs and periphery PCs; however, the CD20low/−CD138+CD31+ panel was specific for BM PCs as it did not stain peak (7 days after a booster immunization) peripheral PCs, which are CD138−. Human BM PCs are either CD19+ or CD19−, with recent reports demonstrating that long-lived PCs are contained in the CD19− compartment (16, 17). In this regard, we note that currently available anti−CD19 Abs, including clone J3-119, resolve rhesus CD19+ and CD19− populations suboptimally. Therefore, we opted to exclude this marker from our panels, except in the experiments where direct comparisons with human BM were performed. In those experiments, we demonstrate that unlike on human cells, both CD20low/−CD138+CD31+ and CD20low/−CD138+CD31− rhesus BM cells stained positive for CD38, suggesting that this marker is also not suitable to define rhesus BM PC.

In addition to CD31, we evaluated the adhesion molecule CD49d and the lymphocyte activation antigen CD98 as markers for rhesus BM PCs. These markers were previously shown to be expressed on human or mouse PCs; CD138, CD31, and CD49d are involved in PCs interactions with the BM microenvironment, and their overexpression is a key feature of healthy human BM PCs (15, 29, 31). CD31 is expressed on a large number of cell types, and it has been used to isolate human PCs from BM (32, 33), tonsils (34), lamina propria (31), and spleen (29), belongs to the Ig gene superfamily, and is the ligand of CD38 (35). CD49d is a transmembrane protein, which associates with integrin beta 1 to form the heterodimer VLA-4 and is a prognostic marker for chronic lymphocytic leukemia (CLL) (36, 37). CD98 is important for activation, proliferation, and effector functions of T and B cells (38, 39) and was recently described as a marker for BM PCs in mice (30). We demonstrate here that CD31 expression correlates strongly with CD49d and CD98 expression on CD20low/−CD138+ rhesus BM cells.

In humans, the distribution of PC-produced Ig isotypes varies in different organs. For instance, IgG predominates in the BM followed by IgA (16, 40), while in the spleen, the distribution is more equal (29), and IgA is most abundant in mucosa-associated lymphoid tissues (41). In our studies, rhesus BM PCs produced primarily IgG (60%), followed by IgA (33%) (16, 40). The presence of IgA-secreting cells in the BM of rhesus and humans is interesting and represents an important area of investigation to complement recent reports of PCs in the gut (40, 42, 43). The relationship between Ab specificities encoded by antigen-specific memory B cells, BM PCs, and circulating Abs is of significant interest for our understanding of vaccine-induced responses. Data from the mouse model suggest that the peripheral memory B cell compartment retains a larger diversity of Ab specificities against a given antigen than does the BM PC compartment (44, 45). In humans, studies have compared the specificities of antigen-specific BM PCs with serum Abs in individuals vaccinated with Tetanus toxoid (46) or infected with HIV-1 (47), but so far rhesus macaques were not used to address such questions despite their frequent use in vaccine evaluation. We also demonstrate a selective loss of CD138+CD31+ cells upon cryopreservation of BM cells using the conditions described in Section “Materials and Methods.” Similarly, others have reported a reduction in the frequency of plasmablasts following cryopreservation of PBMCs, as shown by both by flow cytometry and B cell ELISpot analyses (48). These observations have important implications for studies of the Ab repertoires in these compartments.

In conclusion, we show that functional rhesus macaque BM PCs secreting IgG, IgA, and IgM are highly enriched in the CD20low/−CD138+CD31+ population. These studies offer a foundation for studies aimed at investigating the quality of long-lived Ab responses in an important primate model and an approach for isolating single PCs from the rhesus macaque BM compartment for Ab cloning and transcriptional analyses.

## Author Contributions

PM-M designed, performed, and analyzed the experiments shown in Figures 1, 2, 3, and 6; LP designed, performed, and analyzed the experiments shown in Figures 4 and 5; CS contributed valuable inputs on the results and the manuscript; KH performed the transmission electron microscopy; MS provided the rhesus macaque bone marrow samples; PW provided human bone marrow samples; GH designed the experiments, reviewed the results, and wrote the paper together with PM-M.

## Conflict of Interest Statement

The authors declare that the research was conducted in the absence of any commercial or financial relationships that could be construed as a potential conflict of interest.
