# Supplementary material for: CD138 and CD31 Double-Positive Cells Comprise the Functional Antibody-Secreting Plasma Cell Compartment in Primate Bone Marrow
Source: Front Immunol. 2016 Jun 27;7:242. doi: 10.3389/fimmu.2016.00242 (PMC4921460; doi:10.3389/fimmu.2016.00242)
Supplement: Supplementary file 1 [file Image_1.PDF]

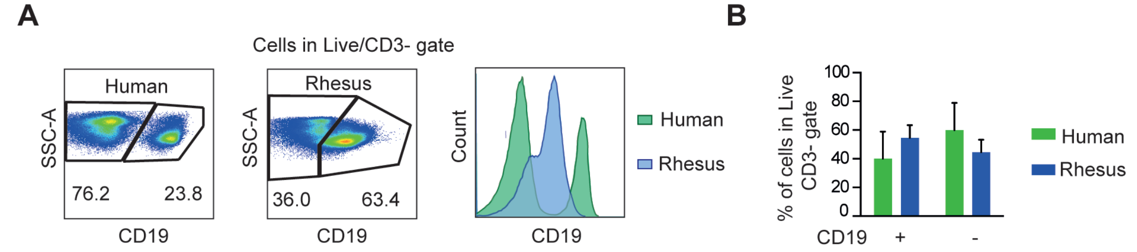

Supplementary Figure 1

(A) Representative flow cytometry data showing human and rhesus bone marrow cells pre-gated for Live/CD3- cells and stained with the anti-CD19 Ab, clone J3-119. Data from representative subjects are shown as dot plots (left) and overlay histograms (center).

(B) Frequencies(mean and SD) of CD19+ and CD19- cells of Live/CD3- cells in rhesus (n=3) and human (n=3) bone marrow are shown as bar diagrams (right).
