# Supplementary material for: CD138 and CD31 Double-Positive Cells Comprise the Functional Antibody-Secreting Plasma Cell Compartment in Primate Bone Marrow
Source: Front Immunol. 2016 Jun 27;7:242. doi: 10.3389/fimmu.2016.00242 (PMC4921460; doi:10.3389/fimmu.2016.00242)
Supplement: Supplementary file 2 [file Image_2.PDF]

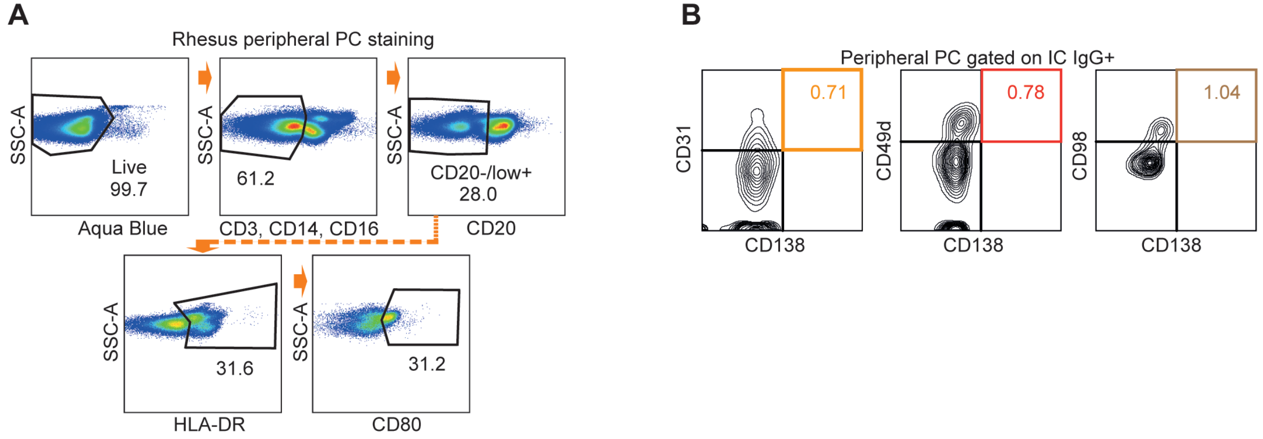

Supplementary Figure 2
(A) Flow cytometry gating strategy for rhesus peripheral blood PCs defined as CD3-CD14-CD16-CD20low/-HLA-DR+CD80+ cells based on Silveira et al. (B) Flow cytometry contour plots showing CD138+CD31+ (orange), CD138+CD49d+ (red) and CD138+CD98+ (brown) populations pre-gated for Live/CD3-/IgG+ cells as shown in Fig 4A using PBMCs collected at day 7 after immunization.
